# Association of A1 Segment Morphology with the Rupture Risk and Morphology of Anterior Communicating Artery Aneurysms: A Retrospective, Single-Center Study

**DOI:** 10.3390/jcm15041376

**Published:** 2026-02-10

**Authors:** Ilhan Aydin, Neslihan Cavusoglu, Berkay Kef, Asya Gokceli, Efecan Cekic, Sahin Hanalioglu, Egemen Gok, Murad Asilturk, Bulent Timur Demirgil

**Affiliations:** 1 Department of Neurosurgery, Istanbul Bakırköy Mazhar Osman Training and Research Hospital for Mental Health and Neurological Disorders, Istanbul 34147, Türkiye; 2 Department of Neurosurgery, Artvin State Hospital, Artvin 08000, Türkiye; neslihancavusoglu.md@gmail.com; 3 Department of Neurosurgery, Biruni University Hospital, Istanbul 34295, Türkiye; bkef@biruni.edu.tr; 4 Department of Neurosurgery, Faculty of Medicine, Hacettepe University, Ankara 06100, Türkiye; drefecancekic@gmail.com (E.C.);; 5 Department of Neurosurgery, Medicana Ataköy Hospital, Istanbul 34158, Türkiye

**Keywords:** anterior communicating artery, A1 segment, intracranial aneurysm, aneurysm rupture, vascular anatomy, cerebral hemodynamics

## Abstract

**Background/Objectives:** A1 segment asymmetry, including hypoplasia and aplasia, is a well-recognized anatomical variation associated with altered hemodynamic stress and anterior communicating artery (ACoA) aneurysm formation. However, its influence on subsequent aneurysm rupture risk remains controversial. This study aimed to evaluate the relationship between A1 segment morphology and aneurysm rupture risk, as well as its association with aneurysm size and morphological complexity. **Methods:** A retrospective single-institution analysis was conducted on 211 patients treated for ACoA aneurysms between June 2016 and March 2025. A1 segment morphology was assessed using digital subtraction angiography and categorized as symmetric, hypoplastic (diameter < 1 mm or <50% of the contralateral vessel), or aplastic. Demographic, clinical, and radiological variables were recorded. Statistical analyses included univariate comparisons with Bonferroni correction for multiple testing and multivariable logistic regression to identify independent predictors of aneurysm rupture. **Results:** The study population had a mean age of 54.72 ± 10.97 years, with a male-to-female ratio of 1.24:1 (55.5% male, 44.5% female). Symmetric A1 segments were observed in 49.3% of patients, hypoplastic segments in 31.3%, and aplastic segments in 19.4%. No statistically significant association was identified between A1 morphology and aneurysm rupture rates (*p* = 0.251) or mean aneurysm diameter (*p* = 0.996). Univariate analysis demonstrated that younger age (*p* = 0.006), male sex (*p* = 0.016), and smoking (*p* = 0.033) were associated with rupture. However, none of these factors, including A1 morphology, remained independent predictors of rupture in the multivariable logistic regression model. **Conclusions:** Although A1 segment asymmetry is common in patients with ACoA aneurysms, it does not independently influence rupture risk or aneurysm morphology. Our findings suggest that rupture behavior is driven primarily by dynamic hemodynamic factors rather than static anatomical variations.

## 1. Introduction

Intracranial aneurysms are abnormal, localized dilatations of cerebral arteries that arise from structural weakening of the vessel wall due to hemodynamic stress and vascular remodeling [1]. Their overall prevalence in the general population is estimated to range from 2% to 5% [2,3]. Aneurysm formation is influenced by a combination of modifiable risk factors, such as cigarette smoking, hypertension, and excessive alcohol consumption, and non-modifiable factors including age, sex, a family history of subarachnoid hemorrhage (SAH), autosomal dominant polycystic kidney disease, and Ehlers-Danlos syndrome type IV [4,5]. Although most intracranial aneurysms remain asymptomatic and are incidentally detected, approximately 1% of them progress to rupture [6]. When rupture occurs, it frequently results in SAH, a life-threatening condition often described by patients as “the worst headache of their life” [1].

Intracranial aneurysms tend to develop at arterial bifurcations, particularly along the convex side of a vascular curve, aligned with the direction of blood flow in the absence of curvature [1]. Their pathogenesis is thought to involve a complex interplay between hemodynamic forces and the biological response of the arterial wall, ultimately leading to focal wall degeneration and weakening [7]. Among these, aneurysms of the anterior communicating artery (ACoA) are particularly notable, accounting for approximately 25% of all intracranial aneurysms and exhibiting a higher propensity for rupture compared to aneurysms located elsewhere in the anterior circulation [7,8,9]. Furthermore, ACoA aneurysms are characteristically smaller in size at the time of rupture. They are associated with worse neurological and neuropsychological outcomes, as well as a higher risk of vasospasm and long-term deficits [10,11,12].

The anterior cerebral circulation demonstrates considerable anatomical variability, particularly in the A1 segment of the anterior cerebral artery (ACA). Variations such as hypoplasia or aplasia create a cross-flow across the ACoA, leading to increased hemodynamic burden and shear stress at the ACoA complex [13,14,15]. While this “hyperdynamic” flow environment is widely accepted as a primary driver of aneurysm formation, its role in precipitating aneurysm rupture remains a subject of persistent controversy. The literature presents conflicting evidence: some studies, such as those by Castro et al. and Park et al., posit that A1 asymmetry significantly increases rupture risk due to elevated wall stress [7,16]. Conversely, other major series, including those by Rinaldo et al. and Zhang et al., have failed to demonstrate a significant correlation between A1 morphology and rupture status, suggesting that static anatomical variations may not predict instability [17,18,19]. This unresolved discrepancy highlights a critical gap in current risk stratification models.

In this retrospective study, we aimed to evaluate whether the configuration of the A1 segment (symmetric vs. hypoplastic/aplastic) is associated with differences in aneurysm morphology and rupture risk in patients diagnosed with ACoA aneurysms.

## 2. Materials and Methods

### 2.1. Patient Selection and Data Collection

This retrospective study included patients who were diagnosed with ACoA aneurysm and treated either with microsurgical clipping or endovascular intervention at our tertiary institution between June 2016 and March 2025. Patient data were retrieved from the hospital’s electronic medical records. Inclusion criteria comprised all patients diagnosed with ruptured or unruptured ACoA aneurysms confirmed via digital subtraction angiography (DSA).

Demographic data, including age, sex, smoking status, and history of hypertension, were recorded. Clinical presentation at admission was categorized as either ruptured or incidental. Radiological evaluation was conducted using DSA to determine maximum aneurysm diameter, presence of a secondary (daughter) sac, and overall aneurysm morphology.

Exclusion criteria were as follows: (1) age under 18 years; (2) presence of central nervous system comorbidities such as tumors or arteriovenous malformations; (3) fusiform, mycotic, dissecting, traumatic, or extradural aneurysms; (4) aneurysms located outside the ACoA region; and (5) absence of high-resolution DSA imaging. A total of 247 patients with ACoA aneurysms were initially screened. Of these, 36 patients were excluded based on the study criteria: 24 due to the absence of high-resolution DSA imaging (relied solely on CTA/MRA), 6 due to non-saccular aneurysm morphology (fusiform, traumatic, or dissecting), 4 due to coexisting CNS pathologies (arteriovenous malformations or tumors), and 2 for being under 18 years of age. Consequently, 211 patients met the inclusion criteria and were analyzed.

### 2.2. Assessment of A1 Segment Morphology

The morphology of the A1 segments was evaluated using DSA images. Segment diameters were measured from their origin at the terminal portion of the internal carotid artery. To minimize measurement bias, assessments were independently performed by two senior interventional radiologists and two experienced neurosurgeons.

The relative diameter criterion (<50%) was the principal measure; the absolute cutoff (<1 mm) was used only to support measurements when both A1 segments were small, in accordance with prior literature [16]. Aplasia was defined as the complete absence of the A1 segment on angiographic imaging. Based on these criteria, patients were classified into two morphological categories: (1) symmetric A1 morphology; (2) asymmetric A1 morphology, including both hypoplastic and aplastic segments.

### 2.3. Statistical Analysis

All statistical analyses were performed using R software (version 4.4.3) with the associated packages (tidyverse, gtsummary, stats). Categorical variables (e.g., aneurysm morphology [symmetric, hypoplastic, aplastic], rupture status, hypertension, smoking) were summarized as counts and percentages. Between-group comparisons were conducted using the Chi-square test or Fisher’s exact test when more than 20% of expected cell counts were <5. Continuous variables (e.g., age, aneurysm size) were presented as mean ± standard deviation (SD). Distributional assumptions were evaluated with the Shapiro–Wilk test. For non-normally distributed variables, the Mann–Whitney U test was used for two-group comparisons. For normally distributed data, the independent-samples *t*-test was used for two-group comparisons, and the one-way ANOVA was used for comparisons across more than two groups.

To address multiple testing, a Bonferroni correction was applied for the pairwise comparisons regarding A1 morphology, setting the statistical significance threshold at *p* < 0.0071 (0.05/7). Additionally, variables identified as significant in the univariate analysis for rupture status (age, sex, smoking, and hypertension) were entered into a multivariable logistic regression model to assess their independent associations with rupture. A1 morphology was excluded from the model because it was not significant in the univariate analysis. The statistical tests were two-tailed, and *p*-values < 0.05 were considered statistically significant.

## 3. Results

### 3.1. Baseline Characteristics and A1 Segment Morphology

**Figure 1:**
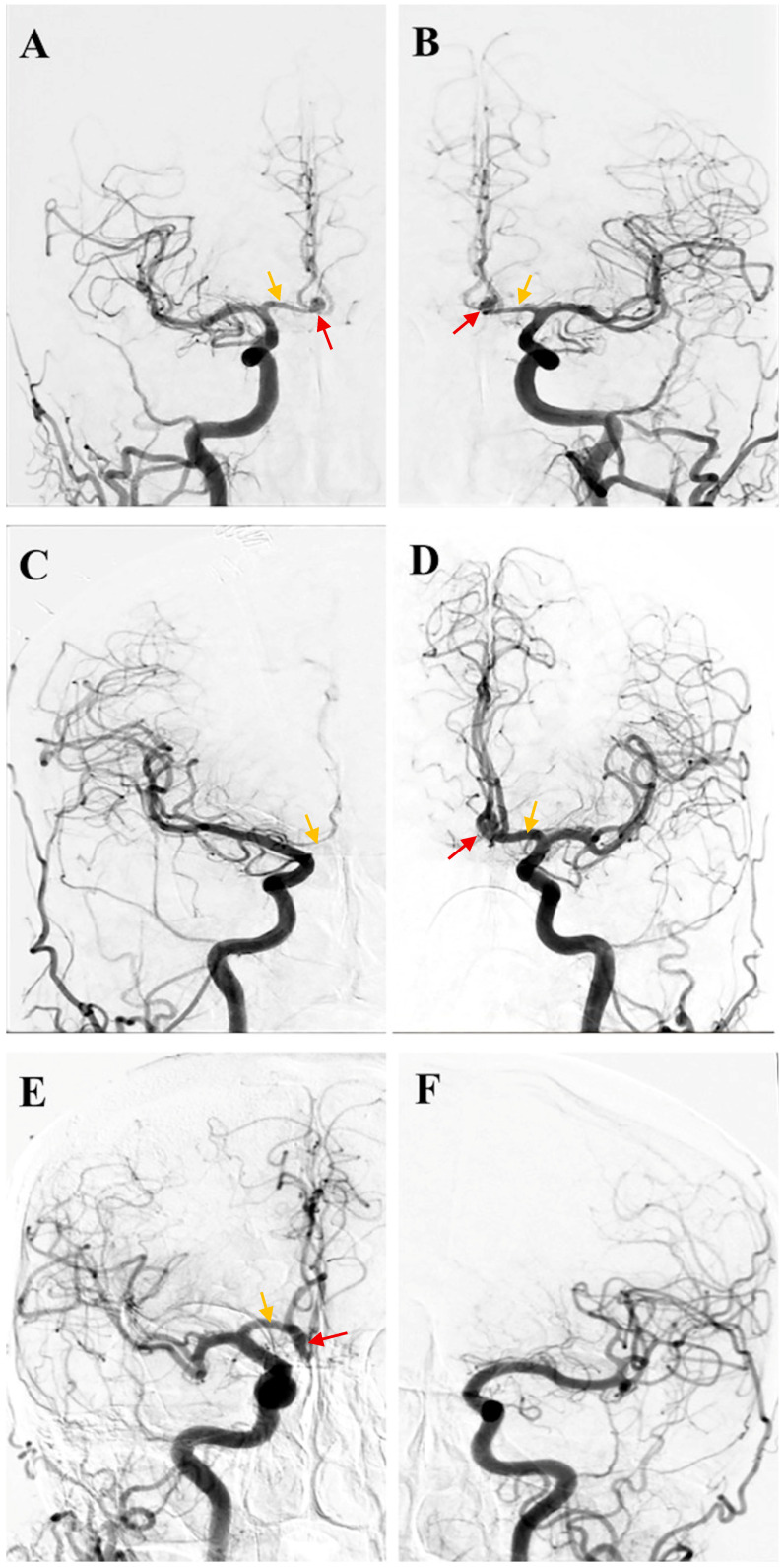
Representative digital subtraction angiography images demonstrating the three A1 segment configurations. (**A**,**B**) Symmetric A1 segments. (**C**,**D**) Hypoplastic A1 segment (diameter < 1 mm and/or <50% of the contralateral vessel). (**E**,**F**) Aplastic A1 segment (complete absence of anterograde flow). Red arrows indicate the aneurysm, while orange arrows denote A1 morphology. Each image pair represents a single subject: panels (**A**,**B**) show symmetric A1 segments; panels (**C**,**D**) illustrate unilateral A1 hypoplasia, with a hypoplastic A1 in panel (**C**) and a normal contralateral A1 in panel (**D**); panels (**E**,**F**) depict unilateral A1 aplasia, with a normal A1 in panel (**E**) and absence of the corresponding A1 in panel (**F**).

**Figure 2:**
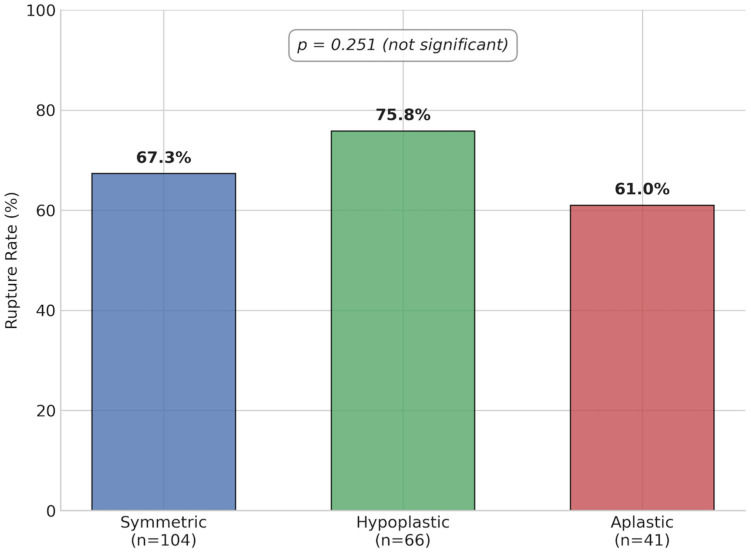
Aneurysm rupture rates according to A1 segment morphology. The percentages represent the rupture rate within each A1 morphology category (symmetric: 67.3%, hypoplastic: 75.8%, aplastic: 61.0%). The difference among groups was not statistically significant (*p* = 0.251).

A total of 211 patients treated for AcoA aneurysms were included in the study. As shown in Figure 1, based on angiographic evaluation, 104 patients (49.3%) had symmetric A1 segments, 66 (31.3%) had A1 hypoplasia, and 41 (19.4%) had A1 aplasia (Figure 2). As detailed in Table 1, the cohort’s mean age was 54.72 ± 10.97 years, with no statistically significant difference among the three morphological groups (*p* = 0.269). Similarly, gender distribution (*p* = 0.726) and hypertension prevalence (*p* = 0.691) were comparable across groups.

Regarding aneurysm morphology, the mean aneurysm radius was 5.50 ± 2.23 mm, and this did not differ significantly between patients with symmetric (5.48 ± 2.31 mm), hypoplastic (5.51 ± 2.30 mm), or aplastic (5.5 ± 1.97 mm) A1 segments (*p* = 0.996). The presence of a secondary (daughter) sac was also not associated with A1 morphology (*p* = 0.536). While smoking prevalence initially appeared higher in the symmetric A1 group compared to the hypoplastic and aplastic groups (60.6% vs. 40.9% and 39.0%, respectively), the associated *p* value (*p* = 0.012) did not surpass the predefined Bonferroni-adjusted threshold (*p* < 0.0071) and was therefore interpreted as a non-significant trend rather than a statistically significant difference.

### 3.2. Analysis of Rupture Risk

Of the 211 patients, 145 (68.7%) presented with a ruptured aneurysm, while 66 (31.3%) were diagnosed incidentally. As detailed in Table 2, univariate analysis identified several demographic and clinical factors associated with rupture status. Patients in the ruptured group were significantly younger than those in the incidental group (53.4 ± 11.2 vs. 57.7 ± 9.8 years, *p* = 0.006). Male sex was more prevalent among ruptured cases (61.4%) compared to incidental cases (42.4%, *p* = 0.016). Additionally, the rate of active smoking was significantly higher in the ruptured group (55.2% vs. 39.4%, *p* = 0.0326). Conversely, a history of hypertension was observed less frequently in patients with ruptured aneurysms (40.0%) compared to those with unruptured aneurysms (59.1%, *p* = 0.015).

Crucially, no statistically significant association was found between A1 segment morphology and aneurysm rupture (*p* = 0.251); rupture rates were 67.3% for symmetric, 75.8% for hypoplastic, and 61.0% for aplastic segments. Aneurysm dimensions and the presence of a secondary sac also showed no significant correlation with rupture status (*p* = 0.493 and *p* = 0.255, respectively).

**Figure 3:**
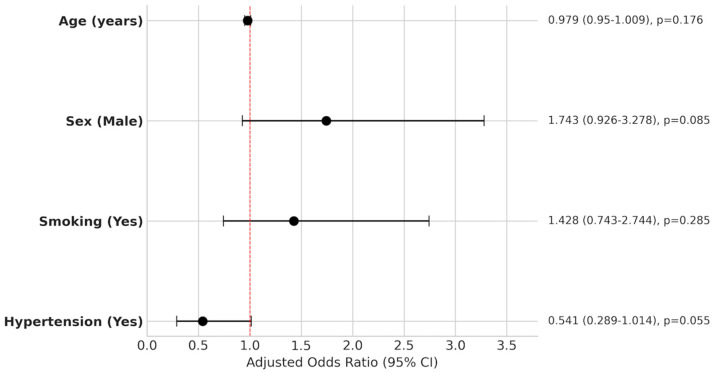
Forest plot of the multivariable logistic regression analysis illustrating the independent association between each covariate and aneurysm rupture, expressed as adjusted odds ratios (aORs) with 95% confidence intervals. The dashed red line indicates an aOR of 1.0. Predictors located to the right of this line indicate a positive association with rupture, while those to the left indicate a negative (protective) association.

As demonstrated in Figure 3, to determine independent predictors of rupture, a multivariable logistic regression analysis was performed, including age, sex, smoking, and hypertension. As detailed in Table 3, none of the variables remained statistically significant independent predictors of rupture: Age (*p* = 0.176; odds ratio [OR] 0.979), Sex (*p* = 0.085; OR 1.743), Smoking (*p* = 0.285; OR 1.428), and Hypertension (*p* = 0.055; OR 0.541).

## 4. Discussion

Our study identified several findings with clinical and theoretical implications. Most notably, no significant association was found between A1 segment morphology and aneurysm rupture. Despite a numerically higher rupture rate in the hypoplastic group, the difference did not reach statistical significance (*p* = 0.251). Likewise, aneurysm characteristics such as mean diameter and the presence of a daughter sac did not differ across A1 morphological types. These results suggest that while A1 asymmetry may influence aneurysm formation through altered hemodynamics, it may not independently drive rupture or alter aneurysmal morphology once the aneurysm is established. The relatively high proportion of ruptured cases (68.7%) likely reflects the referral pattern of our primarily surgical tertiary center, where a larger share of ruptured ACoA aneurysms is treated microsurgically.

Conversely, several patient-related factors demonstrated significant associations with rupture in the univariate analysis. Ruptured aneurysms occurred more frequently in younger patients (mean age 53.4 vs. 57.7 years, *p* = 0.006) and in males (61.4% vs. 42.4%, *p* = 0.016). This is opposing with reports in the general literature favoring female and older age predominance. The high prevalence of smoking in our ruptured cohort (55.2%, *p* = 0.033) may have contributed to accelerated vessel wall degradation, potentially precipitating rupture at a younger age. Additionally, the predominance of young males in the rupture group likely reflects a referral bias inherent to our tertiary surgical center. Younger patients with acute SAH are aggressively transferred for microsurgical clipping, whereas older, asymptomatic patients or those with significant comorbidities may be more frequently managed conservatively or referred for endovascular treatment, leading to their underrepresentation in our surgical series. However, it is important to note that these demographic associations did not persist in the multivariable analysis, suggesting they are likely confounded by other variables.

ACoA aneurysms account for approximately 30–37% of all intracranial aneurysms and are associated with a higher risk of rupture compared to other aneurysms in the anterior circulation [18,20]. According to the hyperdynamic theory, altered hemodynamic stress due to segmental asymmetry, particularly involving the A1 segment, plays a key role in both aneurysm formation and rupture [18,19]. Specifically, the imbalanced flow resulting from A1 segment asymmetry (hypoplasia or aplasia) elevates wall stress at the ACoA complex. While the role of A1 asymmetry in aneurysm formation is well documented in the literature, the extent to which this anatomical variation influences the risk of rupture remains a matter of debate [17,19].

### 4.1. A1 Segment Asymmetry and Aneurysm Formation vs. Rupture

The link between asymmetric inflow at the ACoA complex and aneurysm formation is well established [7,13,14,15]. Hemodynamic stress from imbalanced flow (when one A1 segment is dominant) is widely accepted as a primary driver of aneurysm initiation and growth [7,21,22]. Many studies have demonstrated a higher rate of A1 hypoplasia or aplasia in patients with ACoA aneurysms than in controls or in those with aneurysms at other locations [18,19,21,23]. Although our study was not designed to test the risk of aneurysm formation, the high rate of A1 asymmetry in our group (50.7%) aligns with these previous findings.

Whether this asymmetry increases rupture risk remains controversial. Our results are consistent with studies reporting no such correlation. Rinaldo et al. found no significant difference in A1 hypoplasia between ruptured and unruptured aneurysm groups [17]. Similarly, Zhang et al. found no association in univariate analysis, and Hassanin et al. concluded that A1 anomalies offer “no predictive value for aneurysm rupture” [18,19]. Other studies, however, have reported opposite results. For instance, Park et al. found that a dominant A1 segment was associated with aneurysm rupture (*p* = 0.011) [16]. The mixed evidence suggests that while hemodynamic stress from A1 asymmetry may be sufficient to initiate aneurysm formation, other factors—such as aneurysm shape, wall shear stress, and patient-specific characteristics—may play a greater role in determining rupture risk [7,24].

### 4.2. Impact of A1 Asymmetry on Aneurysm Morphology

Our study found no significant relationship between A1 configuration and aneurysm morphology, specifically mean diameter and the presence of a daughter sac. The mean aneurysm diameters were nearly identical across the symmetric, hypoplastic, and aplastic groups. This result is consistent with the findings of Zhang et al., who also reported no significant difference in aneurysm size between symmetric and asymmetric A1 groups [18]. This contrasts with other reports. Rinaldo et al. found that ACoA aneurysms associated with hypoplastic A1 segments were significantly larger (mean 7.7 mm vs. 6.0 mm) and had a lower dome-to-neck ratio [17]. The authors suggested that altered hemodynamics could directly influence both aneurysm size and shape. The discrepancy between our findings and those of Rinaldo et al. may stem from differences in patient populations, underlying comorbidities, or subtle variations in measurement methodology [17]. The impact of A1 asymmetry on aneurysm growth may not be uniform and may be modulated by other coexisting risk factors.

Furthermore, recent literature suggests that the geometric relationship between the A1 segment and the aneurysm dome may be more predictive of behavior than A1 caliber alone. A 2024 study by Zhang et al. proposed a novel classification based on the axis connecting the A1 origin to the aneurysm base [25]. They demonstrated that the aneurysm’s orientation (e.g., straight, superior, or inferior projection) was significantly associated with rupture presentation (*p* = 0.019) and morphological complexity, such as neck width and height. This supports the notion that detailed vascular geometry and flow angles, likely play a more critical role in rupture mechanics than the static presence of A1 hypoplasia.

### 4.3. Ancillary Findings and Contradictions

In our cohort of patients with ACoA aneurysms, we initially observed that individuals with a hypoplastic A1 segment were significantly less likely to have a history of smoking compared to those with symmetric A1 segments (40.9% vs. 60.6%, *p* = 0.012). This initial observation mirrors the findings of Rinaldo et al., who also reported a significantly lower history of smoking among patients with A1 hypoplasia compared to their counterparts with symmetric A1 anatomy [17]. However, this association is not universally supported in the literature. For instance, a study by Park et al. found no statistically significant difference in smoking history between symmetric and asymmetric (hypoplastic/aplastic) A1 groups [16]. This discrepancy may suggest that variations in population characteristics or study methodologies could influence the observed outcomes. However, no biological or pathophysiological mechanism has been described in the literature by which adult smoking would directly affect the morphology of the A1 segment. Consistent with the lack of a biological basis, after applying the Bonferroni correction for multiple comparisons as specified in our methodology, this finding lost statistical significance. This suggests that the initial association was likely a statistical artifact, specifically Type 1 Error. Nevertheless, we must consider that the relatively limited sample size of our single-center cohort might have lacked the power to detect a weak but true association. Alternatively, unmeasured confounding factors, such as socioeconomic status or genetic traits potentially linking vascular development with addictive behaviors, could have influenced the distribution. However, without a plausible biological mechanism linking a congenital variation (A1 hypoplasia) to an acquired habit (smoking), we interpret the loss of significance after adjustment as validation that no true causal relationship exists.

### 4.4. Determinants of Rupture Risk

Our univariate analysis suggested that younger age, male sex, and smoking were associated with a higher likelihood of rupture, while hypertension appeared protective. However, the multivariable analysis revealed that these factors were not independent predictors of rupture in our cohort. For instance, while hypertension was more prevalent in the unruptured group (*p* = 0.015 in univariate), it showed a borderline non-significant trend in the multivariate model (*p* = 0.055). This counterintuitive finding, where a history of hypertension appeared inversely associated with rupture, likely reflects a treatment effect. Patients with a known diagnosis of hypertension are typically under active pharmacological management and regular monitoring, which maintains their hemodynamic wall stress within a controlled range. Conversely, patients in the ruptured group may include a significant proportion of undiagnosed or non-compliant individuals who are subject to unmitigated blood pressure spikes, which are known triggers for rupture [6,11]. Similarly, the loss of significance for smoking and sex in the multivariate model suggests that these variables may share variance or that the sample size was insufficient to detect more minor independent effects. This highlights the complexity of rupture risk, which is likely driven by a combination of hemodynamic factors (e.g., wall shear stress) and patient-specific factors beyond standard demographic variables.

While our study is based on standard angiographical measurements that are widespread in clinical practice, we must acknowledge that such macroscopic classification (symmetric, hypoplastic/aplastic) might be oversimplifying complex vascular geometry. The lack of a statistical correlation in our study does not rule out the influence of A1 morphology entirely; rather, it suggests that standard linear measurements may be insufficient to capture subtle high-dimensional features associated with rupture risk. Latest advancements in the field of radiomics and machine learning have enabled multi-feature extraction approaches that analyze texture, shape complexity, and intensity heterogeneity. These methods may predict aneurysm instability with greater accuracy than traditional morphology-based evaluation alone [26,27,28]. Future research considering these radiomic features combined with hemodynamic parameters may clarify the relationship between the anterior cerebral complex anatomy and aneurysm behavior that traditional statistical models failed to detect.

## 5. Limitations

Certain limitations of this study must be mentioned. First, the retrospective design introduces inherent risks of bias. Specifically, as a tertiary referral center with a primary focus on microsurgical clipping, our cohort included a high proportion of ruptured cases (68.7%). This likely reflects a referral selection bias, where unruptured aneurysms treated endovascularly or managed conservatively might be underrepresented, potentially limiting the generalizability of our results to the broader population of incidental ACoA aneurysms. Second, as a single-center study, our findings may not be generalizable to populations with different genetic or environmental backgrounds. Third, the absence of longitudinal data in our cross-sectional analysis precludes the assessment of aneurysm growth rates or evolving hemodynamic patterns over time. Fourth, this study relied solely on static morphological data and did not employ advanced post-processing techniques such as radiomics or computational fluid dynamics (CFD). Consequently, high-dimensional quantitative features or dynamic factors like wall shear stress that might predict rupture risk were not assessed. Furthermore, we acknowledge the possibility of residual confounding from unmeasured variables; for instance, specific antihypertensive treatment regimens or detailed genetic predispositions were not available for analysis. Finally, the morphology of a ruptured aneurysm may be altered by the rupture event itself, potentially confounding the analysis of pre-rupture characteristics.

## 6. Conclusions

In conclusion, this study demonstrates that, while A1 segment asymmetry is a critical factor in the formation of ACoA aneurysms, its association with rupture risk and specific morphological features remains uncertain. Our data suggest that in patients with established ACoA aneurysms, the presence of A1 hypoplasia or aplasia does not, in itself, confer a higher risk of rupture or result in larger or more complex aneurysms. This suggests that, once an aneurysm has formed, its subsequent behavior may be governed by distinct morphological and hemodynamic factors.

Future research should prioritize prospective, multicenter studies to overcome the limitations of retrospective analyses. Integrating advanced imaging techniques, particularly patient-specific CFD, will be crucial to move beyond static anatomical descriptions and directly quantify the hemodynamic forces hypothesized to drive aneurysm growth and rupture. Such studies will help clarify existing controversies and refine risk stratification for patients with unruptured ACoA aneurysms.

## Figures and Tables

**Table 1:** Baseline Characteristics of Patients Operated on for Anterior Communicating Artery (ACoA) Aneurysms.

	Symmetrical A1 (n = 104)	Hypoplasia in A1 (n = 66)	Aplasia in A1 (n = 41)	Total (n = 211)	*p*-Value
**Age (years)**	53.93 ± 11.10	56.53 ± 10.88	53.78 ± 10.69	54.72 ± 10.97	0.269
**Sex**					0.726
Male	59 (56.7%)	34 (51.5%)	24 (58.5%)	117 (55.5%)	
Female	45 (43.3%)	32 (48.5%)	17 (41.5%)	94 (44.5%)	
**Smoking**					0.012
No	41 (39.4%)	39 (59.1%)	25 (61.0%)	105 (49.8%)	
Yes	63 (60.6%)	27 (40.9%)	16 (39.0%)	106 (50.2%)	
**Aneurysm diameter (mm)**	5.48 ± 2.31	5.51 ± 2.30	5.50 ± 1.97	5.50 ± 2.23	0.996
**Secondary Sac**					0.536
No	76 (73.1%)	43 (65.2%)	28 (68.3%)	147 (69.7%)	
Yes	28 (26.9%)	23 (34.8%)	13 (31.7%)	64 (30.3%)	
**Hypertension**					0.691
No	59 (56.7%)	33 (50.0%)	22 (53.7%)	114 (54.0%)	
Yes	45 (43.3%)	33 (50.0%)	19 (46.3%)	97 (46.0%)	
**Ruptured Aneurysm**					0.251
No	34 (32.7%)	16 (24.2%)	16 (39.0%)	66 (31.3%)	
Yes	70 (67.3%)	50 (75.8%)	25 (61.0%)	145 (68.7%)	

**Table 2:** Comparison of Demographic and Clinical Characteristics Between Patients with Ruptured and Unruptured Anterior Communicating Artery (ACoA) Aneurysms.

	Ruptured Aneurysm (n = 145)	Unruptured Aneurysm (n = 66)	*p*-Value
**Age (years)**	53.4 ± 11.2	57.7 ± 9.8	0.006
**Sex**			0.016
Male	89 (61.4%)	28 (42.4%)	
Female	56 (38.6%)	38 (57.6%)	
**Smoking**			0.033
No	65 (44.8%)	40 (60.6%)	
Yes	80 (55.2%)	26 (39.4%)	
**Aneurysm diameter (mm)**	5.57 ± 2.11	5.33 ± 2.50	0.493
**Secondary Sac**			0.255
No	97 (66.9%)	50 (75.8%)	
Yes	48 (33.1%)	16 (24.2%)	
**Hypertension**			0.015
No	87 (60.0%)	27 (40.9%)	
Yes	58 (40.0%)	33 (59.1%)	
**A1 Morphology**			0.251
Symmetrical	70 (48.3%)	34 (51.5%)	
Hypoplasia	50 (34.5%)	16 (24.2%)	
Aplasia	25 (17.2%)	16 (24.2%)	

**Table 3:** Multivariable Logistic Regression Analysis of Factors Associated with Rupture.

	aOR (95% CI)	*p*-Value
Age (years)	0.979 (0.950–1.009)	0.176
Sex (Male vs. Female)	1.743 (0.926–3.278)	0.085
Smoking (Yes vs. No)	1.428 (0.743–2.744)	0.285
Hypertension (Yes vs. No)	0.541 (0.289–1.014)	0.055

Abbreviations: aOR, adjusted Odds Ratio; CI, Confidence Interval.

## Data Availability

The data presented in this study are available on reasonable request from the corresponding author. The data are not publicly available due to privacy and ethical restrictions related to patient information during the study.

## Abbreviations

The following abbreviations are used in this manuscript:

ACoA	Anterior communicating artery
SAH	Subarachnoid hemorrhage
ACA	Anterior cerebral artery
DSA	Digital subtraction angiography
SD	Standard deviation
aOR	Adjusted odds ratio
CI	Confidence interval
CFD	Computational Fluid Dynamics
